# Does perinatal management have the potential to reduce the risk of intraventricular hemorrhage in preterm infants?

**DOI:** 10.3389/fped.2024.1361074

**Published:** 2024-01-31

**Authors:** Peter Korček, Jan Širc, Ivan Berka, Jáchym Kučera, Zbyněk Straňák

**Affiliations:** ^1^ Neonatal Intensive Care Unit, Institute for the Care of Mother and Child, Prague, Czech Republic; ^2^ Third Faculty of Medicine, Charles University, Prague, Czech Republic

**Keywords:** perinatal management, preterm infants, intraventricular hemorrhage, mode of delivery, antenatal steroids, neonatal morbidity, early onset sepsis

## Abstract

**Background:**

Intraventricular hemorrhage (IVH) is an important cause of neurodevelopmental impairment in preterm infants. A number of risk factors for IVH have already been proposed; however, some controversies regarding optimal perinatal management persist. This study aimed to identify perinatal and neonatal attributes associated with IVH in a representative population of preterm infants.

**Methods:**

Perinatal data on 1,279 very preterm infants (<32 weeks of gestation) admitted to a tertiary neonatal intensive care unit were analyzed. The records were assessed using univariate analysis and logistic regression model to evaluate the risk factors for any and high–grade IVH (grade III–IV according to the classification by Papile) within the first week after birth.

**Results:**

The incidence of any IVH was 14.3% (183/1,279); the rate of low–grade (I–II) and high–grade (III–IV) IVH was 9.0% (115/1,279) and 5.3% (68/1,279), respectively. Univariate analysis revealed multiple factors significantly associated with intraventricular hemorrhage: lower gestational age and birth weight, absence of antenatal steroids, vaginal delivery, low Apgar score at 5 min, delivery room intubation, surfactant administration, high frequency oscillation, pulmonary hypertension, pulmonary hemorrhage, tension pneumothorax, persistent ductus arteriosus, hypotension and early onset sepsis. Logistic regression confirmed lower gestational age, vaginal delivery, ductus arteriosus and early onset sepsis to be independent predictors for any IVH. Pulmonary hemorrhage, tension pneumothorax and early onset sepsis were independent risk factors for high–grade IVH. Complete course of antenatal steroids was associated with a lower risk for any (odds ratio 0.58, 95% confidence interval 0.39–0.85; *P* = .006) and for high–grade intraventricular hemorrhage (odds ratio 0.36, 95% confidence interval 0.20–0.65; *P* < .001).

**Conclusion:**

The use of antenatal steroids and mode of delivery are crucial in the prevention of IVH; however, our study did not confirm the protective effect of placental transfusion. Severe respiratory insufficiency and circulatory instability remain to be powerful contributors to the development of IVH. Early detection and management of perinatal infection may also help to reduce the rate of brain injury and improve neurodevelopment in high–risk newborns.

## 1 Introduction

Intraventricular hemorrhage (IVH) is one of the most common types of brain injury in very preterm (<32 weeks of gestation) infants (1). The immature central nervous system is highly susceptible to the development of IVH due to undeveloped vasculature in the germinal matrix and periventricular white matter (2). Furthermore, the preterm brain is very sensitive to hypoxia–ischemia owing to low baseline cerebral blood flow, high oxygen consumption and increased oxygen extraction (3). Thus, cardio–respiratory instability and dysfunctional cerebral autoregulation can lead to IVH in the early postnatal period (4).

The majority of IVH events occur within the first 72 h after birth and may progress during the first postnatal week (5). The presence and severity of IVH significantly correlates with mortality and neurodevelopmental impairment, therefore, it is essential to properly identify factors associated with IVH in preterm infants (6).

Multiple risk factors have already been confirmed for the development of IVH (i.e., outborn patients, low gestational age and birth weight), however, uncertainty persists for many others (chorioamnionitis, mode of delivery, early onset sepsis, and the severity of respiratory distress syndrome including its complications) (7–9). In contrast, pregnancy–induced hypertension, tocolytic therapy, antenatal steroids, indomethacin or surfactant administration have been associated with reduced incidence of IVH (9–11). Recently published data have shown some protective role of placental transfusion (especially using delayed cord clamping) on frequency of IVH (12).

The aim of our study was to analyze perinatal factors associated with the development of IVH including specific obstetric and neonatal procedures in very preterm infants.

## 2 Materials and methods

### 2.1 Study design and participants

**Figure 1:**
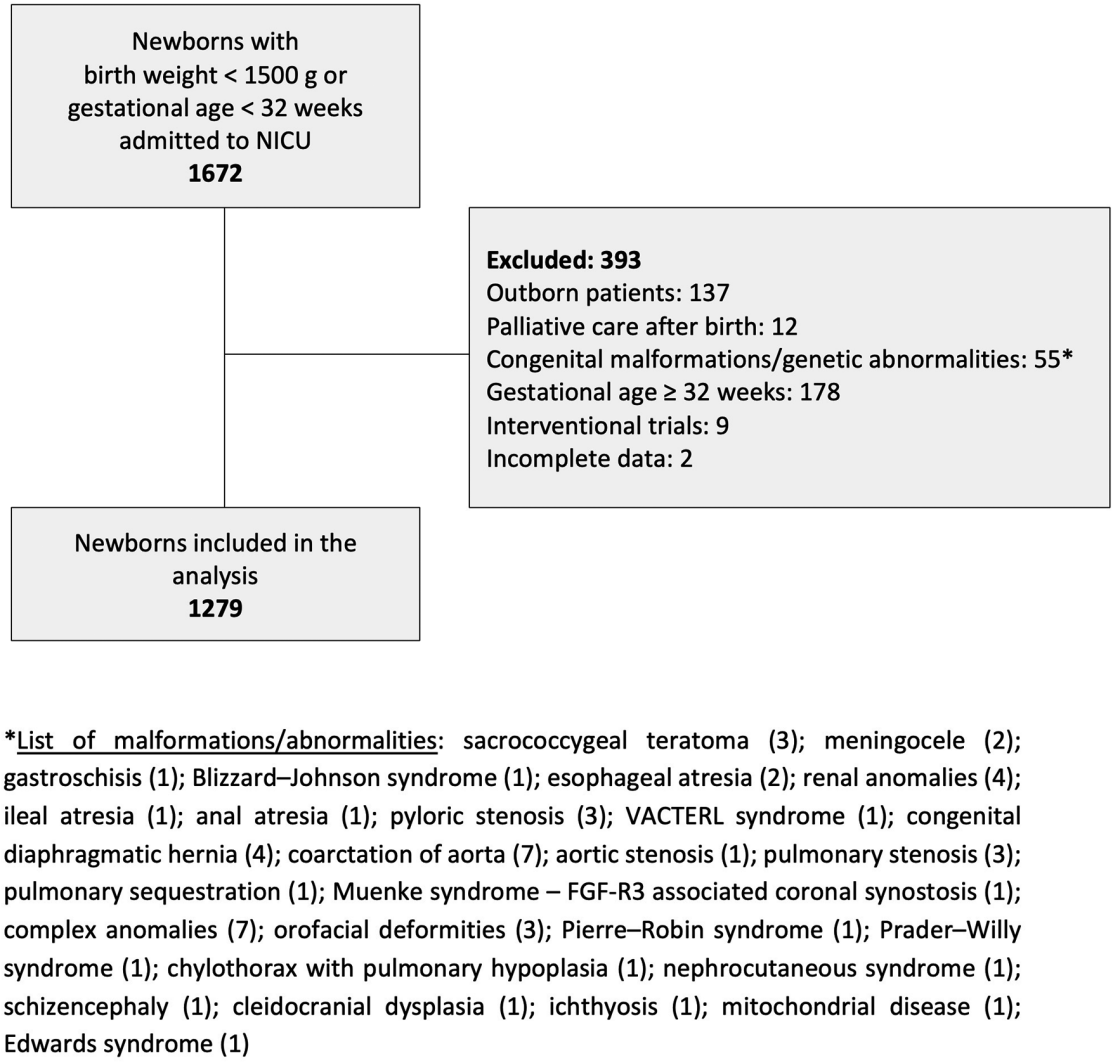
Flowchart of the study design.

This was a retrospective analysis of prospectively collected data of very preterm (<32 weeks of gestation) and very low birth weight (VLBW) infants (BW < 1,500 g) admitted to a tertiary neonatal intensive care unit between January 2013 and December 2022. Perinatal and neonatal data were collected using the standardized datasheet. The study exclusion criteria were: outborn birth; palliative care after birth; serious congenital and chromosomal abnormalities; participation in interventional trials; gestational age (GA) ≥ 32 weeks of gestation and incomplete data (Figure 1). The study was performed in accordance with the principles outlined in the Declaration of Helsinki and approved by the Institutional Review Board and Ethics Committee. Written informed consent to collect and use the anonymized data was provided by the participants' legal guardians.

### 2.2 Perinatal characteristics

Gestational age was determined as the best obstetric estimate using ultrasonography and/or date of the last menstrual period. Neonatal anthropometric characteristics were obtained in accordance with Fenton growth charts (2013–2015) and Intergrowth-21 (2016–2022); small–for–gestational–age (SGA) infants were considered those with BW <10th percentile for gender and GA (13, 14). Furthermore, data on multiple pregnancy (including monochorionic/diamniotic twins), mode of delivery and prenatal care were included. Dexamethasone (*Dexamed by Medochemie, Limassol, Cyprus*) was used as an antenatal corticosteroid of choice at a dose of 6 mg administered intramuscularly every 12 h (four doses in total) (15). Fetal therapy involved amniodrainage and/or laser coagulation of pathologic placental anastomoses in a monochorionic pregnancy complicated by twin–twin transfusion syndrome or twin anemia polycythemia sequence (16). Placental transfusion was defined as either delayed cord clamping (DCC ≥ 30–60 s) or umbilical cord milking (UCM; approximately 20 cm of the cord milked 3–5 times by a delivering obstetrician) (17). Initial temperature (axilla) was obtained by a standard neonatal thermometer following the admission of a newborn to the unit.

### 2.3 Neonatal characteristics

Following morbidities and therapies within the first week of life were considered for the analysis. Respiratory distress syndrome (RDS) was treated with non–invasive ventilation support (Continuous Positive Airway Pressure—CPAP) or surfactant administration (*Curosurf by Chiesi, Parma, Italy*) (18). Invasive mechanical ventilation at any time was defined as positive pressure ventilation through the endotracheal tube with respective ventilatory settings—conventional ventilation or high frequency oscillatory ventilation (HFOV). Caffeine citrate (*Peyona by Chiesi, Parma, Italy*) at loading dose of 20 mg/kg and maintenance dose at 5–10 mg/kg/day was administered as early after birth as possible to support spontaneous breathing on nasal CPAP or prior to elective extubation. Tension pneumothorax was defined as extrapleural air diagnosed by radiograph and requiring chest tube placement. The diagnosis of pulmonary hemorrhage was made when hemorrhagic secretions were aspirated from the trachea concomitant with ventilation decompensation that required intubation or intensified cardiorespiratory support (19).

Persistent pulmonary hypertension of a newborn (PPHN) was documented whenever inhaled nitric oxide (10–20 ppm) or Sildenafil was used: 1–2 mg/kg/dose every 6 h orally or intravenous form at dose of 1.6 mg/kg/day (*Revatio by Pfizer, New York, USA*). Systemic hypotension was defined as mean arterial blood pressure ≤ GA in weeks and requiring intervention: volume therapy (10–20 ml/kg of Plasmalyte), inotropic drugs (Dopamine 2–20 µg/kg/min, Noradrenaline 0.1–1.0 µg/kg/min, Dobutamine 2–40 µg/kg/min) and Hydrocortisone in vasopressor–resistant scheme of 1 mg/kg/dose every 8 h for 2–3 days). Hemodynamically significant patent ductus arteriosus (PDA) was diagnosed using targeted neonatal echocardiography and treatment based on the clinical course and ultrasound findings. Initial treatment consisted of drugs: Paracetamol (*Paracetamol Kabi by Fresenius Kabi, Hamburg, Germany*) 60 mg/kg/day for 4–6 days or Ibuprofen (*Pedea by Recordati S.*p*.*A*., Milan, Italy*) 10 mg/kg followed by a dose of 5 mg/kg 24 and 48 h later. Open surgery technique (ligaclip or ligature) was performed in selected infants at a later stage.

Early onset sepsis (EOS) was defined as occurring within 72 h after birth and by using the following criteria: positive culture (blood, cerebrospinal fluid) within 48 h from specimen collection and the presence of clinical signs of infection; or a clinical sepsis with negative culture (blood, cerebrospinal fluid), present clinical signs of infection and serum C-reactive protein level >10 mg/L. The following were considered as clinical signs of infection: hypothermia, respiratory instability (apnea, desaturations, respiratory distress syndrome with ongoing mechanical ventilation), cardiovascular (cyanosis, bradycardia, poor peripheral perfusion, hypotension) and neurological (lethargy, suspected seizures) symptoms.

### 2.4 Intraventricular hemorrhage

Cranial ultrasound examinations were performed through the anterior fontanelle by trained and experienced attending neonatologists using ultrasound diagnostic systems (*Philips CX50/HD15/Epiq5 by Philips Ultrasound, Inc., Washington, USA*). A pediatric radiologist was consulted in case the diagnosis of IVH was less clear. The first cranial ultrasound was performed within 72 h after birth and there were at least two scans made during the first week of life. Severity of IVH was evaluated according to the Papile system: grade I—subependymal hemorrhage limited to germinal matrix; grade II—primary IVH without ventricular dilatation; grade III—IVH with ventricular dilatation; grade IV—IVH with intraparenchymal involvement (periventricular hemorrhagic infarction). Grades I and II were considered mild or low–grade IVH, while grades III and IV were considered severe or high–grade IVH (20). The most severe grade of IVH diagnosed during the first week of life was included in the statistical analysis.

### 2.5 Statistical analysis

Newborns were grouped and compared based on the incidence of any and high–grade IVH. Continuous data were expressed as the mean ± standard deviation and categorical data as counts and rate (%). Comparisons between the groups were performed using the *χ*^2^ test or Fisher's exact test for categorical variables and independent samples *T*–test for continuous variables. All significant variables in the univariate analysis were subsequently included in the multivariable logistic regression model, and the independent risk factors of IVH and its severity were determined accordingly. Omnibus test was used to test whether the explained variance in a set of data is significantly greater than the unexplained variance. The Hosmer and Lemeshow test was used to determine the goodness of fit for the logistic regression model. The statistical tests were two–sided with *P* < .05 being statistically significant. The analysis was performed with IBM SPSS Statistics for Windows (*SPSS 28.0; SPSS Institute, Chicago, IL, USA*).

## 3 Results

### 3.1 Population characteristics

Of the 1,279 infants included in the analysis, 712 (55.7%) were males and 567 (44.3%) were females (male–to–female ratio 1.25:1). The mean gestational age and birth weight was 28.3 ± 2.2 weeks and 1,132 ± 362 grams, respectively. ANS were administered completely in 972 (76.0%) newborns and partially in 248 (19.4%) newborns; 59 (4.6%) newborns did not receive any ANS. Cesarean section was performed in 1,033 (80.8%) cases and mean Apgar score at 5 min and admission temperature were 7.6 ± 1.3 and 36.6 ± 0.5 °C, respectively. A total of 1,233 (96.4%) infants had RDS and 649 (50.7%) infants received surfactant. Overall, 260 (20.3%) newborns were treated for hypotension during the first 72 h and EOS was present in 122 (9.5%) cases. Mortality rate was 5.9% (76 infants).

### 3.2 Intraventricular hemorrhage

**Figure 2:**
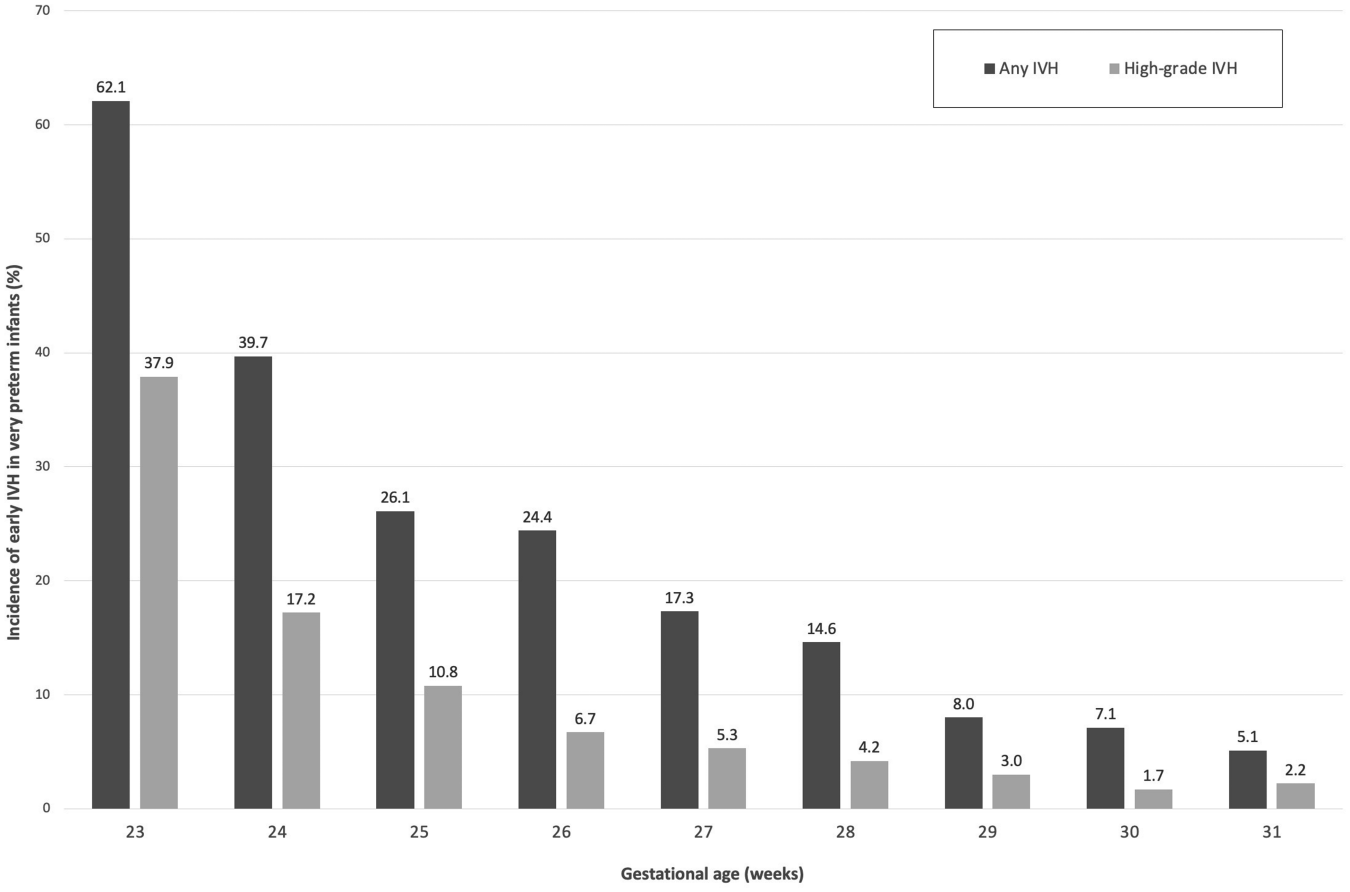
Rate of early (first week after birth) intraventricular hemorrhage (IVH) in very preterm infants based on gestational age at birth.

Overall incidence of any IVH in the population was 14.3% (183/1,279), while the incidence of low–grade (I–II) and high–grade (III–IV) IVH was 9.0% (115/1,279) and 5.3% (68/1,279), respectively. The relative occurrence of IVH with respect to GA can be seen in Figure 2.

The incidence of any IVH in the 23–25, 26–28, and 29–31 weeks' subgroups were 35.4% (70/198), 18.0% (66/367), and 6.6% (47/714), respectively, with significant difference observed among the subgroups (*P* < .001). The incidence of any IVH based on BW was 23.3% (118/507), 8.4% (48/574), and 8.6% (17/198) for <1,000, 1,000–1,499, and ≥1,500 g, respectively, with significant difference observed among the subgroups (*P* < .001).

The incidence of high–grade IVH in the 23–25, 26–28, and 29–31 weeks' subgroups were 16.7% (33/198), 5.2% (19/367), and 2.2% (16/714), respectively, with significant difference observed among the subgroups (*P* < .001). The incidence of high–grade IVH based on BW was 9.5% (48/507), 2.3% (13/574), and 3.5% (7/198) for <1,000, 1,000–1,499, and ≥1,500 g, respectively, with significant difference observed among the subgroups (*P* < .001).

Univariate analysis revealed risk factors significantly associated with any IVH (Table 1) and high–grade IVH (Table 2): lower GA and BW, absence of ANS, vaginal delivery, low Apgar score at 5 min, delivery room intubation, surfactant administration, caffeine citrate underuse, high frequency oscillatory ventilation, pulmonary hypertension, pulmonary hemorrhage, tension pneumothorax, PDA requiring therapy, hypotension and EOS. In contrast, fetal therapy, monochorionic pregnancy, prenatal care, male gender, SGA status at birth, placental transfusion, admission temperature and RDS had no influence on the occurrence of IVH.

**Table 1:** Comparison between no IVH and any IVH subgroups using univariate analysis.

Variables	No IVH (*n* = 1,096)	Any IVH (*n* = 183)	*p*-value
Gestational age (weeks)	28.7 ± 2.1	26.7 ± 2.4	<.001
29–31	667 (60.9%)	47 (25.7%)	
26–28	301 (27.4%)	66 (36.0%)	
23–25	128 (11.7%)	70 (38.3%)	
Birth weight (grams)	1,165 ± 354	935 ± 354	<.001
≥1,500	181 (16.5%)	17 (9.3%)	
1,000–1,499	526 (48.0%)	48 (26.2%)	
<1,000	389 (35.5%)	118 (64.5%)	
Fetal therapy	62 (5.7%)	13 (7.1%)	.400
Monochorionic pregnancy	245 (22.4%)	31 (16.9%)	.120
Prenatal care	1,081 (98.6%)	181 (98.9%)	1.000
Antenatal steroids (complete)	856 (78.1%)	116 (63.4%)	<.001
Male gender	608 (55.5%)	104 (56.8%)	.748
Small for gestational age	159 (14.5%)	29 (15.8%)	.652
Vaginal delivery	180 (16.4%)	66 (36.1%)	<.001
Multiple birth	527 (48.1%)	68 (37.2%)	.006
Placental transfusion	615 (56.1%)	103 (56.2%)	1.000
Delayed cord clamping	438 (40.0%)	72 (39.3%)	
Umbilical cord milking	177 (16.1%)	31 (16.9%)	
Apgar score at 5 min	7.7 ± 1.2	7.0 ± 1.6	<.001
Delivery room intubation	180 (16.4%)	87 (47.5%)	<.001
Admission temperature (°C)	36.6 ± 0.5	36.6 ± 0.7	.340
Respiratory distress syndrome	1,054 (96.2%)	179 (97.8%)	.389
Caffeine citrate	1,024 (93.4%)	161 (88.0%)	.014
Surfactant administration	520 (47.4%)	129 (70.5%)	<.001
INSURE	84 (7.7%)	5 (2.7%)	
LISA	122 (11.1%)	9 (4.9%)	
Continuous Ventilation	314 (28.6%)	115 (62.9%)	
HFOV	80 (7.3%)	56 (30.6%)	<.001
PPHN	30 (2.7%)	20 (10.9%)	<.001
Pulmonary hemorrhage	45 (4.1%)	26 (14.2%)	<.001
Tension pneumothorax	21 (1.9%)	9 (4.9%)	.029
PDA treatment	66 (6.0%)	35 (19.1%)	<.001
Drugs	47 (4.3%)	23 (12.5%)	
Ligation	13 (1.2%)	4 (2.2%)	
Drugs + Ligation	6 (0.5%)	8 (4.4%)	
Hypotension treatment	179 (16.3%)	81 (44.3%)	<.001
Volume	81 (7.4%)	14 (7.7%)	
Volume + Inotropes	44 (4.0%)	24 (13.1%)	
Volume + Inotropes + Steroids	54 (4.9%)	43 (23.5%)	
Early onset sepsis	84 (7.7%)	38 (20.8%)	<.001
Mortality	34 (3.1%)	42 (23.0%)	<.001

HFOV, high frequency oscillatory ventilation; INSURE, INtubation-SURfactant-Extubation; IVH, intraventricular hemorrhage; LISA, less invasive surfactant administration; PDA, persistent ductus arteriosus; PPHN, persistent pulmonary hypertension of newborn.

**Table 2:** Comparison between no/low–grade IVH and high–grade IVH subgroups using univariate analysis.

Variables	No/low–grade IVH (*n* = 1,211)	High–grade IVH (*n* = 68)	*p*-value
Gestational age (weeks)	28.5 ± 2.2	26.3 ± 2.6	<.001
29–31	698 (57.6%)	16 (23.5%)	
26–28	348 (28.7%)	19 (27.9%)	
23–25	165 (13.6%)	33 (48.5%)	
Birth weight (grams)	1,146 ± 357	885 ± 382	<.001
≥1,500	191 (15.8%)	7 (10.3%)	
1,000–1,499	561 (46.3%)	13 (19.1%)	
<1,000	459 (37.9%)	48 (70.6%)	
Fetal therapy	71 (5.9%)	4 (5.9%)	1.000
Monochorionic pregnancy	262 (21.6%)	14 (20.6%)	1.000
Prenatal care	1,194 (98.6%)	68 (100.0%)	1.000
Antenatal steroids (complete)	937 (77.4%)	35 (51.5%)	<.001
Male gender	674 (55.7%)	38 (55.9%)	1.000
Small for gestational age	173 (14.3%)	15 (22.1%)	.110
Vaginal delivery	223 (18.4%)	23 (33.8%)	.004
Multiple birth	566 (46.7%)	29 (42.6%)	.534
Placental transfusion	681 (56.2%)	37 (54.4%)	.802
Delayed cord clamping	483 (39.9%)	27 (39.7%)	
Umbilical cord milking	198 (16.3%)	10 (14.7%)	
Apgar score at 5 min	7.7 ± 1.2	6.7 ± 1.8	<.001
Delivery room intubation	226 (18.7%)	41 (60.3%)	<.001
Admission temperature (°C)	36.6 ± 0.5	36.5 ± 0.8	.127
Respiratory distress syndrome	1,168 (96.4%)	65 (95.6%)	.732
Caffeine citrate	1,135 (93.7%)	50 (73.5%)	<.001
Surfactant administration	594 (49.1%)	55 (80.9%)	<.001
INSURE	89 (7.4%)	0 (0.0%)	
LISA	127 (10.5%)	4 (5.9%)	
Continuous ventilation	378 (31.2%)	51 (75.0%)	
HFOV	110 (9.1%)	26 (38.2%)	<.001
PPHN	43 (3.6%)	7 (10.3%)	.014
Pulmonary hemorrhage	55 (4.5%)	16 (23.5%)	<.001
Tension pneumothorax	24 (2.0%)	6 (8.8%)	.004
PDA treatment	89 (7.3%)	12 (17.6%)	.008
Drugs	61 (5.0%)	9 (13.2%)	
Ligation	15 (1.2%)	2 (2.9%)	
Drugs + Ligation	13 (1.1%)	1 (1.5%)	
Hypotension treatment	222 (18.3%)	38 (55.9%)	<.001
Volume	89 (7.3%)	6 (8.8%)	
Volume + Inotropes	58 (4.8%)	10 (14.7%)	
Volume + Inotropes + Steroids	75 (6.2%)	22 (32.4%)	
Early onset sepsis	106 (8.8%)	16 (23.5%)	<.001
Mortality	45 (3.7%)	31 (45.6%)	<.001

HFOV, high frequency oscillatory ventilation; INSURE, INtubation-SURfactant-Extubation; IVH, intraventricular hemorrhage; LISA, less invasive surfactant administration; PDA, persistent ductus arteriosus; PPHN, persistent pulmonary hypertension of newborn.

### 3.3 Independent risk factors for any IVH

Factors significantly associated with any IVH in univariate analysis were included in the multivariable logistic regression model (Table 3). Lower GA, vaginal delivery, PDA and EOS were found to be independent risk factors for any IVH, whereas a complete course of ANS was associated with a lower risk for any IVH (*P* = .006).

**Table 3:** Logistic regression analysis of independent variables associated with any IVH.

Parameter	B	SE	Wald *Χ*^2^	OR (95% CI)	*p*-value
Gestational age	−0.191	0.073	6.781	0.82 (0.71–0.95)	.009
Birth weight	0.000	0.000	0.009	1.00 (0.99–1.00)	.926
Antenatal steroids (complete)	–0.539	0.197	7.470	0.58 (0.39–0.85)	.006
Vaginal delivery	0.846	0.224	14.216	2.32 (1.50–3.61)	<.001
Multiple birth	–0.124	0.200	0.383	0.88 (0.59–1.30)	.536
Apgar score at 5 min	–0.079	0.077	1.070	0.92 (0.79–1.07)	.301
Delivery room intubation	0.274	0.275	0.992	1.31 (0.76–2.25)	.319
Caffeine citrate	–0.293	0.323	0.824	0.74 (0.39–1.40)	.364
Surfactant administration	–0.342	0.257	1.772	0.71 (0.42–1.17)	.183
HFOV	0.522	0.290	3.250	1.68 (0.95–2.97)	.071
PPHN	0.408	0.378	1.168	1.50 (0.71–3.15)	.280
Pulmonary hemorrhage	0.287	0.316	0.827	1.33 (0.71–2.47)	.363
Tension pneumothorax	0.558	0.473	1.389	1.74 (0.69–4.41)	.239
PDA	0.619	0.275	5.064	1.85 (1.08–3.18)	.024
Hypotension treatment	0.330	0.246	1.804	1.39 (0.85–2.25)	.179
Early onset sepsis	0.796	0.257	9.572	2.21 (1.33–3.67)	.002

HFOV, high frequency oscillatory ventilation; IVH, intraventricular hemorrhage; PDA, persistent ductus arteriosus; PPHN, persistent pulmonary hypertension of newborn.

The significance value of the Omnibus test (model coefficients) indicated the current model outperformed the null model (*χ*^2^ = 188.163, *P* < .001). The Hosmer and Lemeshow test (*χ*^2^ = 4.538, *P* = .806) indicated the model fit the data well. Specificity (true negative rate) and sensitivity (true positive rate) using the current model was 98.4% and 22.4%, respectively. Overall, the model accurately classified 87.5% of any IVH cases.

### 3.4 Independent risk factors for high–grade IVH

Factors significantly associated with high–grade IVH in univariate analysis were included in the multivariable logistic regression model (Table 4). Pulmonary hemorrhage, tension pneumothorax and EOS were found to be independent risk factors for severe IVH, whereas a complete course of ANS was associated with a lower risk for severe IVH (*P* < .001). Vaginal delivery was borderline significant for increased risk of high–grade IVH (*P* = .057).

**Table 4:** Logistic regression analysis of independent variables associated with high–grade IVH.

Parameter	B	SE	Wald *Χ*^2^	OR (95% CI)	*p*-value
Gestational age	−0.109	0.119	0.845	0.89 (0.71–1.13)	.358
Birth weight	0.000	0.001	0.040	1.00 (0.99–1.00)	.842
Antenatal steroids (complete)	–1.007	0.296	11.554	0.36 (0.20–0.65)	<.001
Vaginal delivery	0.619	0.325	3.628	1.85 (0.98–3.51)	.057
Apgar score at 5 min	–0.033	0.106	0.097	0.96 (0.78–1.19)	.756
Delivery room intubation	0.483	0.421	1.315	1.62 (0.71–3.70)	.251
Caffeine citrate	–1.518	0.383	15.694	0.21 (0.10–0.46)	<.001
Surfactant administration	0.052	0.459	0.013	1.05 (0.42–2.58)	.910
HFOV	0.152	0.397	0.147	1.16 (0.53–2.53)	.701
PPHN	–0.326	0.542	0.361	0.72 (0.25–2.08)	.548
Pulmonary hemorrhage	1.028	0.396	6.748	2.79 (1.28–6.07)	.009
Tension pneumothorax	1.224	0.552	4.919	3.40 (1.15–10.03)	.027
PDA	0.198	0.406	0.237	1.21 (0.55–2.70)	.626
Hypotension treatment	0.507	0.362	1.968	1.66 (0.81–3.37)	.161
Early onset sepsis	0.857	0.363	5.577	2.35 (1.15–4.80)	.018

HFOV, high frequency oscillatory ventilation; IVH, intraventricular hemorrhage; PDA, persistent ductus arteriosus; PPHN, persistent pulmonary hypertension of newborn.

The significance value of the Omnibus test (model coefficients) indicated the current model outperformed the null model (*χ*^2^ = 121.625, *P* < .001). The Hosmer and Lemeshow test (*χ*^2^ = 2.713, *P* = .951) indicated the model fit the data well. Specificity (true negative rate) and sensitivity (true positive rate) using the current model was 99.6% and 13.2%, respectively. Overall, the model accurately classified 95.0% of high–grade IVH cases.

## 4 Discussion

### 4.1 Key findings

Our study confirmed the protective role of ANS on IVH development. C-section seems to be beneficial in the prevention of IVH, whereas no advantage was found in the use of placental transfusion. The severity of respiratory insufficiency and circulatory instability as well as perinatal infection remain crucial in the pathogenesis of IVH in very preterm infants.

### 4.2 Results in the context of what is known

Our results align with other studies that found significant association between early IVH and lower GA and BW (7, 9). However, we found that lower GA (in contrast to BW) is an independent risk factor for any IVH. This finding supports known morphological and functional patterns of developing brain (i.e., highly vascularized germinal matrix, fragile thin-walled capillaries and dysfunctional cerebrovascular autoregulation) (3, 4, 21). Antenatal dexamethasone exerts complex protective effects on vulnerable cerebral vessels (i.e., vasoconstriction, pericyte recruitment, membrane stabilization), including downregulation of growth factors (vascular endothelial growth factor, angiopoietin) responsible for robust angiogenesis in the germinal matrix and increased vascular fragility (15, 22). In agreement with other studies, ANS were found to be independently and significantly associated with reduced risk of any (*P* = .006) and high–grade IVH (*P* < .001) (10, 15).

Our study confirmed the impact of early postnatal complications and interventions (i.e., respiratory insufficiency requiring invasive ventilation, systemic hypotension requiring inotropic agents) on the development of IVH (4, 7, 8). Recently published data showed some benefits of placental transfusion on the respiratory and circulatory disturbances irrespective of the applied technique (DCC or UCM) (12, 23). Moreover, cord milking in preterm infants (<32 weeks of gestation) born by C-section was found to be superior in improving systemic blood flow (higher blood pressure, increased urine output) compared to DCC, but another study from Katheria et al. showed that UCM compared to DCC was associated with a higher rate of severe IVH in extremely preterm newborns (12, 24). A secondary analysis of the latter study revealed that IVH was more likely to occur following vaginal birth compared to cesarean delivery (12). We observed only an association between IVH and the mode of delivery, but no protective effect of placental transfusion (12, 25). These findings might be explained by inconsistency in performing placental transfusion (e.g.,: duration of DCC, number of strippings for UCM), however, these details had not been analyzed in our study due to small sample size.

In our study, almost 81% of preterm infants were born by C-section and the overall incidence of IVH was relatively low in comparison to other studies (21). Furthermore, vaginal birth was independently associated with any IVH (*P* < .001) and borderline significant for severe IVH (*P* = .057). This finding is in harmony with other studies documenting the protective effect of C-section in extremely preterm infants (<26–28 weeks of gestation) (26, 27). Nevertheless, further research is required to clarify if C-section may reduce cerebral blood flow variability and risk of IVH in this highly vulnerable population.

Neonatal infection was the only risk factor significantly associated with any grade of IVH which indicates a powerful impact of systemic inflammatory response on developing brain (i.e., capillary leak syndrome, accelerated oxygen consumption, cytokine-induced neurotoxicity) (28). Moreover, EOS and pro–inflammatory mediators are associated with circulatory instability (i.e., hypotension, myocardial hypocontractility, persistence of the arterial duct) necessitating invasive ventilation or cardiovascular support, thus greatly increasing the odds for IVH (4, 21, 28, 29).

Complex antenatal care is necessary for high-risk pregnancies in order to identify and address risk factors contributing to preterm birth and development of IVH (21, 30). Transfer *in utero* (to a tertiary center), prevention of preterm birth, administering ANS (especially <28 weeks of gestation) and managing perinatal infection remain essential interventions to reduce perinatal mortality and morbidity (21, 28, 30). In our study, ANS and EOS were the only attributes significantly changing the odds for IVH. These findings align with published meta–analyses where steroids were beneficial in reducing severe IVH despite the underlying perinatal infection (31, 32).

### 4.3 Strengths and limitations

Prospectively collected data (using standardized datasheet) on a large number of inborn preterm infants is the strength of our study. Furthermore, we performed logistic regression analysis to find out independent risk factors for IVH in this population. However, conducting a retrospective analysis at a single center, it can be challenging to adopt these findings, even though the incidence of IVH was relatively low.

## 5 Conclusion

Antenatal steroids and the mode of delivery play an essential role in preventing IVH. Nevertheless, our study did not verify the protective impact of placental transfusion. The presence of severe respiratory insufficiency and circulatory instability continues to be significant factors contributing to IVH. Early identification and effective management of perinatal infection and its complications may contribute to lowering the incidence of brain injury and improving neurodevelopmental outcome.

## Data Availability

The raw data supporting the conclusions of this article will be made available by the authors, without undue reservation.
